# Association of Self-Reported Medication Adherence with Potentially Inappropriate Medications in Elderly Patients: A Cross-Sectional Pilot Study

**DOI:** 10.3390/ijerph17165940

**Published:** 2020-08-16

**Authors:** Motoyasu Miyazaki, Masanobu Uchiyama, Yoshihiko Nakamura, Koichi Matsuo, Chika Ono, Miwa Goto, Ayako Unoki, Akio Nakashima, Osamu Imakyure

**Affiliations:** 1 Department of Pharmaceutical and Health Care Management, Faculty of Pharmaceutical Sciences, Fukuoka University, Fukuoka 814-0180, Japan; ko1matsuo@fukuoka-u.ac.jp (K.M.); anakashima@fukuoka-u.ac.jp (A.N.); 2 Department of Pharmacy, Fukuoka University Chikushi Hospital, Chikushino 818-8502, Japan; muchiyama@fukuoka-u.ac.jp (M.U.); chi.bass@icloud.com (C.O.); mgoto1213@fukuoka-u.ac.jp (M.G.); ayakoga@fukuoka-u.ac.jp (A.U.); 3 Department of Emergency and Critical Care Medicine, Faculty of Medicine, Fukuoka University, Fukuoka 814-0180, Japan; pdmxy827@yahoo.co.jp; 4 Department of Pharmacy, Oita Nakamura Hospital, Oita 870-0022, Japan

**Keywords:** elderly patients, polypharmacy, potentially inappropriate medication, self-reported medication adherence, visual analogue scale

## Abstract

Background: Polypharmacy (PP) and potentially inappropriate medications (PIMs) cause problematic drug-related issues in elderly patients; however, little is known about the association between medication adherence and PP and PIMs. This study evaluated the association of self-reported medication adherence with PP and PIMs in elderly patients. Methods: A cross-sectional pilot study was conducted using data collected from electronic medical records of 142 self-administering patients aged ≥65 years, excluding emergency hospitalization cases. Self-reported medication adherence was assessed using the visual analogue scale (VAS). Results: Of the 142 patients, 91 (64.1%) had PP and 80 (56.3%) used at least one PIM. In univariate analysis, patients with a VAS score of 100% had a significantly higher number of female patients and ≥1 PIM use compared to other patients. We found no association between the VAS score and PP. In multivariable analysis, the use of PIMs was significantly associated with a VAS score of 100% (odds ratio = 2.32; 95% confidence interval = 1.16–4.72; *p* = 0.017). Conclusions: Use of PIMs by elderly patients is significantly associated with self-reported medication adherence. Pharmacists should pay more attention to prescribed medications of self-administering elderly patients in order to improve their prescribing quality.

## 1. Introduction

In Japan, the demand for drug therapy for elderly patients (age ≥ 65 years) is increasing with an increase in the number of the elderly [1]. However, the pharmacokinetics and drug responsiveness in elderly patients differ from those in younger adult patients because of aging-related physiological changes, and interactions among drugs administered to treat multiple comorbidities might cause adverse drug events (ADEs) [2,3]. Polypharmacy (PP), the use of five or more medications, is a major problem in terms of increasing risk of ADEs in elderly patients [4,5,6,7,8,9,10,11]. Onoue et al. (2018), in a nationwide retrospective study using 240 million pharmacy claim data items, reported that 69.0% of elderly patients are PP(+) [9]. In addition to ADEs, PP is also associated with an increased risk of falls [4,12], flails [12,13], longer hospitalization [14], drug–drug interactions [15], medication non-adherence [16,17], higher medical costs [18], and mortality [12] in elderly patients.

Potentially inappropriate medications (PIMs), defined as medications with more harmful than beneficial effects on elderly patients [19], are closely related to PP [8,20,21,22,23,24,25,26,27] and are a relevant public health concern for elderly patients [28,29]. In 2015, the Japan Geriatric Society (JGS) updated the “Guidelines for Medical Treatment and its Safety in the Elderly” and proposed medication lists regarding PIMs, “Screening Tool for Older Persons’ Appropriate Prescriptions for Japanese” (STOPP-J), to contribute to improving prescription quality [30,31]. PIMs, similar to PP, are associated with an increased risk of ADEs [27,32,33,34,35], which increases with age [23].

Although medication non-adherence is an important drug-related issue in all populations, it is particularly problematic for elderly patients, who often experience a variety of medical conditions and use more medications compared to other patients [36]. The medication adherence of patients is assessed by evaluating medical/dispensing records and pharmacy claims data, such as the Medication Possession Ratio [37,38] and the Proportion of Days Covered [39,40,41,42]; using electronic monitoring devices [43,44]; using patient self-reports [45], such as the Morisky Medication Adherence Scale [46,47] and the visual analogue scale (VAS) [48,49,50,51,52,53]. The VAS is a tool that helps clinicians or pharmacists assess the medication adherence of patients in routine clinical practice. The VAS has moderate concordance with the claim-based measure in patients taking antidiabetes and lipid-modifying drugs [48], and there is a significant association between the VAS score and anticoagulation control in patients who were treated with warfarin [49]. Selinger et al. (2019) assessed four adherence measurement tools, including VAS, Medication Adherence Report Scale, Medication Possession Ratio, and blood level of thiopurine metabolites in inflammatory bowel disease (IBD) patients and found that all four methods significantly associated with each other [54]. In some of the previous studies, a cut-off value of 80% was used to divide medication adherence into good or poor [48,49,50,51], while other studies used a cut-off value of 100% [52,53]. The VAS is quick, inexpensive, and easily applicable during medical visits and has been validated against pharmacy refills for patients with chronic diseases [48,49,50,51,52,53]. Because we believe that VAS is more advantageous than other tools from this point of view, we have incorporated it into our clinical practice.

Although medication non-adherence among elderly patients might be due to forgetfulness, cognitive decline, or physical inability to self-administer medications [36], the fundamental reasons leading to medication non-adherence vary among patients. In Japan, where the number of the elderly is increasing, it is important for elderly patients to properly manage their medications themselves, including PP or PIMs. However, little is known about the association between self-reported medication adherence and PP or PIMs in self-administering elderly patients in Japan. This study evaluated the association of self-reported medication adherence using the VAS with PP and PIMs in self-administering elderly patients.

## 2. Materials and Methods

### 2.1. Study Setting and Patients

We conducted a cross-sectional pilot study in the Fukuoka University Chikushi Hospital (Chikushino, Japan). The study subjects were patients admitted to the hospital between 1 September 2019, and 29 February 2020. Pharmacists interviewed patients soon after they were admitted to the hospital on weekdays. The questions involved prescribed drugs, over-the-counter (OTC) drug use, history of ADEs, and medication adherence (i.e., VAS). The pharmacists interviewed ~20 patients/day for 5–10 min per patient and recorded the interviews in electronic medical records (EMRs). Most pharmacists were irregularly engaged in this work, but one pharmacist (Ph. X) did this every day.

After the patient completed the hospitalization procedure, the administrative department requested the pharmacist to interview the patient, and the patient was randomly selected. To minimize selection bias (i.e., to ensure the consistency of the method of recording the interview with the patient), we targeted only patients interviewed by Ph. X. Patients < 65 years old, admitted for an emergency, unable to self-medicate, or not taking oral medications were excluded. Finally, we included 142 elderly patients in this study.

The study was approved by the Fukuoka University Medical Ethics Review Board (C20-04-002).

### 2.2. Patient Characteristics and Medication Information

Patients’ data were collected on admission. Patient characteristics and medication information were obtained from their EMRs. Patient characteristics included sex, age, height, body weight, body mass index, and history of ADEs. Medication information included OTC drug use, possession of prescription records, one-dose package (ODP) dispensing, the number, type, and duration of prescribed oral medications, and the number of consulting medical institutions. VAS data were obtained from the pharmacist’s records in the EMRs. At the time of the interview, a pharmacist asked each patient, “What percentage of your medications did you take exactly as your doctor prescribed them?” to evaluate the adherence for all medications. The VAS tool was presented to each patient with a continuous line ranging from 0% to 100%, and he or she was asked to mark the line at his or her best guess about self-medication adherence. A VAS score of 100% was defined as full medication adherence, as described previously [52,53].

As recommended by the JGS, the patients were divided into the following two groups: pre-old (65–74 years) and old (≥75 years) [30].

The list of target drugs, target patient populations, and recommendations developed by the JGS, available on the JGS web page for the STOPP-J in Japanese [55], was used for PIM screening [31]. The list used in this study was shown in Table S1. We checked the administration period of the prescribed oral medications by using patients’ prescription records or patient referral documents scanned and saved in EMRs; however, we could not obtain accurate data on the long-term combined use of multiple antithrombotic agents. Thus, the recommended administration period of the combined use of multiple antithrombotic agents was excluded from screening. We used the Kyoto Encyclopedia of Genes and Genomes drug database (https://www.kegg.jp/kegg/drug/) to search and classify the generic names of medications each patient was taking.

### 2.3. Statistical Analysis

Binary variables were expressed as proportions, while continuous variables were expressed as medians and interquartile ranges (IQRs). Differences in continuous variables between groups were evaluated using the Wilcoxon rank-sum test, and differences in categorical variables were evaluated using the chi-square test or Fisher’s exact test. We divided our study population into two groups based on their medication adherence (VAS): full medication adherence group (i.e., VAS score of 100%) and non-full medication adherence group (i.e., VAS score < 100%). We then compared the patient characteristics and medication information, including PP and PIMs, between them. Factors associated (*p* < 0.1) with a VAS score of 100% in univariate analysis were included in multivariable logistic regression analysis. Age group, history of ADEs, PP, and consulting medical institutions (two or more institutions) were also included in multivariable logistic regression analysis, because these factors are generally associated with medication adherence [36]. All statistical analyses were performed using JMP^®^ 14 (SAS Institute Inc., Cary, NC, USA). *p* < 0.05 was considered statistically significant.

## 3. Results

### 3.1. Patient Characteristics and Medication Information

Table 1 summarizes patient characteristics and medication information. The median (IQR) age was 74 (70–79) years. Of the 142 patients, 55 (38.7%) were female, 74 (52.1%) were pre-old, and 68 (47.9%) were old. In addition, >90% of the patients possessed their prescription records. The median (IQR) number of prescribed oral medications per patient was 5 (3–7) and consulting medical institutions per patient was 1 (1–2). Of the 142 patients, 91 (64.1%) were prescribed ≥5 medications, while 51 were PP(−), and 52 (36.6%) consulted ≥2 medical institutions. In addition, 80 (56.3%) patients used ≥ 1 PIM, while 62 were PIM(−). Among the PIMs, sleeping drugs showed the highest prevalence (23.2%), followed by antidiabetes drugs (17.6%), antithrombotic drug combinations (11.3%), and nonsteroidal anti-inflammatory drugs (9.2%).

### 3.2. VAS Distribution for Self-Reported Medication Adherence

**Figure 1:**
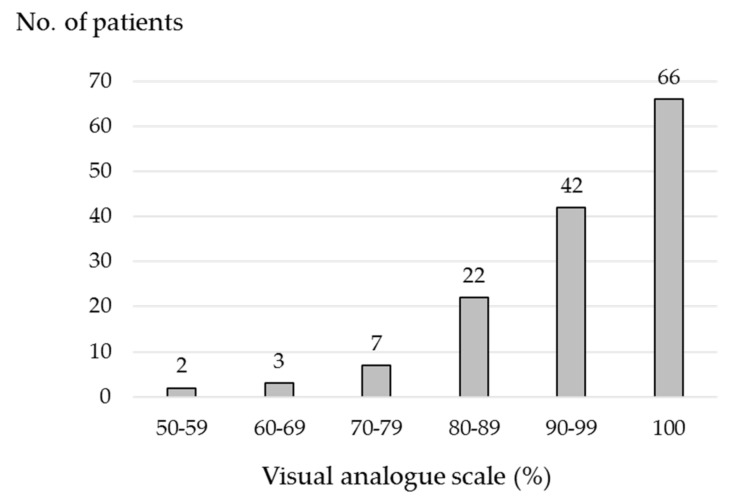
Histogram of the number of patients stratified by 10% of the VAS. VAS, visual analogue scale.

Figure 1 shows the patient distribution stratified by 10% of the VAS. The median (IQR) VAS score was 98% (90–100%), with 66 (46.5%) patients having a VAS score of 100%. Of the remaining 76 patients, 42 (29.6%) had a VAS score of 90–99%; 22 (15.5%), 80–89%; 7 (4.9%), 70–79%; 3 (2.1%), 60–69%; 2 (1.4%), 50–59%. No patients were < VAS 50%.

### 3.3. Comparisons of PP, PIM Use, and VAS between Pre-Old and Old Group

Table 2 shows the results of comparisons of PP, PIM use, and median (IQR) VAS scores between the pre-old and old group. Patients in the old group had a significantly higher number of PP(+) patients compared to the patients in the pre-old group (76.5% vs. 52.7%, *p* = 0.003).

### 3.4. Association of PP with Patient Characteristics and Medication Information

In univariate analysis comparing patient characteristics and medication information for PP(+) and PP(−) patients (Table 3), we found a statistically significant difference in the age group (*p* = 0.003), ODP dispensing (*p* = 0.003), number of consulting medical institutions (*p* = 0.002), and PIM use (*p* = 0.001).

### 3.5. Association of PIMs with Patient Characteristics and Medication Information

In univariate analysis comparing PIM(+) and PIM(−) patients (Table 4), we found a statistically significant difference in sex (*p* = 0.037), ODP dispensing (*p* = 0.020), number of prescribed oral medications (*p* < 0.001), and number of consulting medical institutions (*p* = 0.045).

### 3.6. Factors Associated with Full Medication Adherence in Univariate and Multivariable Analyses

Table 5 shows the results of univariate and multivariable analyses for a VAS score of 100%. In univariate analysis, patients with a VAS score of 100% had a significantly higher number of female patients and ≥1 PIM use compared to patients with a VAS score < 100% (*p* = 0.060 and 0.008, respectively). Multivariable analysis showed that PIM use (odds ratio [OR] = 2.58; 95% confidence interval [CI] = 1.20–5.72; *p* = 0.015) was significantly associated with a VAS score of 100%—that is, full medication adherence.

## 4. Discussion

Self-reported medication adherence is a practical method of measuring a patient’s medication adherence because it is quick and cheap and has the potential to be easily implemented into the clinical workflow. In our hospital, since 1 September 2019, pharmacists have measured the VAS score of hospitalized patients at admission as routine work and, as a result, have provided better medication counseling to these patients than before. The VAS is used to assess medication adherence and shows high median or mean scores in a variety of populations: patients taking antidiabetes (median, 95.9%) and lipid-modifying (median, 95.2%) drugs [48]; hypertension/type 2 diabetes mellitus/dyslipidemia patients (mean, 91.3%) [51]; patients taking at least one hypertensive medication (median, 100%) [56]; IBD, including ulcerative colitis or Crohn’s disease, patients (median, 91–100%) [50,54,57,58]; rheumatoid arthritis patients taking methotrexate (median, 94%) [59]; patients taking warfarin (mean, 92.2–96.6%) [49,60]; patients admitted to the psychiatric ward (mean, 86%) [61]; glaucoma patients (median, 95.0%) [62]; postmenopausal women with hormone receptor–positive breast cancer taking aromatase inhibitors (median, 100%) [63]; human immunodeficiency virus patients undergoing antiretroviral therapy (94–100%) [53,64]. Our median VAS score of 98% was high, similar to previous studies, probably because the study participants were self-administering elderly patients who were highly motivated to take their medications, leading to a high VAS score.

PP and PIMs are important, closely drug-related issues, especially in elderly patients. To the best of our knowledge, this is the first study to evaluate the association of self-reported medication adherence with PP and PIMs in elderly patients in Japan. PP is generally defined as regular use of multiple drugs and is associated with an increased risk of ADEs, hospital admission, and mortality, especially in elderly patients [12,65,66,67,68]. PP is prevalent in elderly patients in Japan. In a nationwide retrospective study using 240 million pharmacy claims data items, Onoue et al. (2018) reported that 69.0% of elderly patients were PP(+) [9]. Ishizaki et al. (2020) also reported that non-excessive PP (5–9 medications) and excessive PP (≥10 medications) was seen, respectively, in 45.3% and 18.2% elderly patients (≥75 years old) [7]. In our study (as shown in Table 2), old (≥75 years) patients had a significantly higher number of PP(+) patients as compared to pre-old (65–74 years) patients (76.5% vs. 52.7%, *p* = 0.003), and 64.1% PP(+) patients had a higher median age (75 years) compared to PP(–) patients (72 years), which is supported by previous studies [7,9].

In our study, ODP dispensing was also associated with PP. PP leads to medication non-adherence [16,17], and ODP dispensing prevents the chance of the unintentional missing of doses, promoting patient safety, and improves medication adherence [69]. In addition, the number of consulting medical institutions was associated with PP and PIM use. Cross-sectional studies in Japan have reported that patients who were prescribed by two or more physicians or who consulted more medical institutions are more likely to have PP and PIM use [8,70], as shown in our study. Furthermore, our study demonstrated that there is also a significant association between PP and PIM use, which is supported by previous reports [8,20,21,22,23,24,25,26,27]. The frequency of elderly PIM(+) patients in Japan varies from 22.9% to 67.3% [8,22,23,27,31,35,68,71,72] because of different study populations and settings, different definitions of PIMs, or different timings of the investigation.

Any PIM use was significantly associated with self-reported full medication adherence in elderly patients. The proportion of PIMs increases with increasing age [23], and medication non-adherence is problematic for elderly patients [36], which leads us to hypothesize that patients taking any PIM are non-adherent to medication; however, our result indicates the opposite. This is probably because we selected elderly patients who can self-administer their medications as study patients. The drug list in STOPP-J is a “List of Medications That Require Particularly Careful Administration,” so patients who can self-administer their medications might have been educated and proactively checked by a physician or pharmacists to prevent ADEs or worsening outcomes. A previous study on medication adherence in atrial fibrillation patients taking direct oral anticoagulants reported a higher adherence of PP in elderly patients compared to younger patients [47]. Our study participants might have been highly motivated regarding their medications, as shown by their VAS scores. Patients who can self-administer their medications and have good medication adherence need to start drug therapy carefully and should be carefully monitored to avoid ADEs caused by continuous use of PIMs.

This study had a few limitations. First, this was a pilot study conducted in a single university hospital, and the study cohort was relatively small. Therefore, our findings might not be generalized to other hospitals or countries. In the future, a multicenter study is necessary to obtain enough sample sizes of the patients. Second, the VAS is a subjective adherence measurement tool and has never been validated in Japan. We defined a VAS score of 100% as a self-reported full medication adherence, as previously described [52,53]; however, previous studies have used a cut-off value of 80% to divide medication adherence into good or poor [48,49,50,51]. The evaluation of medication adherence from the perspective of a pharmacist and the application of an objective measurement tool could not be performed in this study. There are several methods of assessing medication adherence; however, there is no gold standard for measuring adherence [73]. The triangulation of methods is recommended to increase the validity and reliability of the adherence data collected [73]. Further studies are needed to evaluate the relationship between the VAS and other methods, such as administrative claims or electronic pill monitoring in Japan. Third, because of the retrospective study design, we did not examine clinical factors involved in medication adherence. Many potential factors might affect medication adherence, such as education level, severity and duration of illness, patients’ understanding and beliefs about their illness, and medical cost [36]. Lastly, 64.1% and 56.3% patients had polypharmacy and at least one PIM, respectively, but these percentages may be difficult to compare to the previous reports, because we excluded individuals admitted for an emergency or unable to self-medicate. In previous studies which examined individuals using PP and PIMs, it is likely that the incidence rate of unplanned re-admissions and advanced cognitive impairment are associated with PP and PIMs [67,72]. Furthermore, we did not examine the administration period of the combined use of multiple antithrombotic agents, which may have been overestimated in the percentage of PIMs.

## 5. Conclusions

To the best of our knowledge, this is the first study to evaluate the association of self-reported medication adherence with PP and PIMs in a limited elderly patient population excluding emergency hospitalization cases in Japan. PP and PIM prevalence was not uncommon, and self-reported medication adherence was extremely high in elderly patients who can self-administer their medications. There was a significant association between PIM prescription and self-reported full medication adherence. Pharmacists should pay more attention to prescribed medications of self-administering elderly patients in order to improve their prescribing quality.

## Figures and Tables

**Table 1:** Patient characteristics and medication information (*n* = 142).

	*N* (%) or Median (IQR)
Patient characteristics	
Female sex	55 (38.7)
Age (year old) ^a^	74 (70–79)
Pre-old	74 (52.1)
Old	68 (47.9)
Body mass index (kg/m^2^) ^a^	23 (21–25)
History of ADEs	24 (16.9)
Medication information	
OTC drug use	42 (29.6)
Prescription records	131 (92.3)
ODP dispensing	18 (12.7)
No. of prescribed oral medications ^a^	5 (3–7)
Non-polypharmacy	51 (35.9)
Polypharmacy	91 (64.1)
No. of consulting medical institutions	
One institution	90 (63.4)
Two or more institutions	52 (36.6)
No. of patients with PIM use	80 (56.3)
1 PIM	55 (38.7)
2 PIMs	18 (12.7)
3 PIMs	7 (4.9)
Category of PIMs	
Sleeping drugs	33 (23.2)
Sulpiride	1 (0.7)
Antithrombotic drugs (combination)	16 (11.3)
High-ceiling diuretics	8 (5.6)
Alpha 1 blocking agents	2 (1.4)
H_2_ receptor antagonists	7 (4.9)
Drugs for constipation	5 (3.5)
Antidiabetes drugs	25 (17.6)
Overactive bladder drugs	2 (1.4)
NSAIDs	13 (9.2)

^a^ Median (IQR). IQR, interquartile range; ADE, adverse drug event; OTC, over the counter; ODP, one-dose package; PIM, potentially inappropriate medication; NSAIDs, nonsteroidal anti-inflammatory drugs.

**Table 2:** Comparisons of PP, PIM use, and VAS between pre-old and old group (*n* = 142).

	Pre-Old	Old	*p*-Value
	(*n* = 74)	(*n* = 68)	
No. of patients with PP	39 (52.7)	52 (76.5)	0.003
No. of patients with PIM use	43 (58.1)	37 (54.4)	0.657
VAS score ^a^	98 (90–100)	98 (90–100)	0.881

^a^ Median (IQR). PP, polypharmacy; PIM, potentially inappropriate medication; VAS, visual analogue scale; IQR, interquartile range.

**Table 3:** Association of PP with patient characteristics and medication information (*n* = 142).

	PP(+) Patients	PP(−) Patients	*p*-Value
	(*n* = 91)	(*n* = 51)	
Patient characteristics			
Female sex	38 (41.8)	17 (33.3)	0.323
Age (year old) ^a^	75 (71–81)	72 (68–77)	0.005
Pre-old	39 (42.9)	35 (68.6)	0.003
Old	52 (57.1)	16 (31.4)	
Body mass index (kg/m^2^) ^a^	23 (21–25)	24 (21–25)	0.130
History of ADEs	14 (15.4)	10 (19.6)	0.519
Medication information			
OTC drug use	26 (28.6)	16 (31.4)	0.726
Prescription records	84 (92.3)	47 (92.2)	>0.999 ^b^
ODP dispensing	17 (18.7)	1 (2.0)	0.003 ^b^
No. of consulting medical institutions			
One institution	49 (53.8)	41 (80.4)	0.002
Two or more institutions	42 (46.2)	10 (19.6)	
No. of patients with PIM use	63 (69.2)	17 (33.3)	<0.001

^a^ Median (IQR). ^b^ Fisher’s exact test. PP, polypharmacy; ADE, adverse drug event; OTC, over the counter; ODP, one-dose package; PIM, potentially inappropriate medication; IQR, interquartile range.

**Table 4:** Association of PIM use with patient characteristics and medication information (*n* = 142).

	PIM(+) Patients	PIM(−) Patients	*p*-Value
	(*n* = 80)	(*n* = 62)	
Patient characteristics			
Female sex	37 (46.3)	18 (29.0)	0.037
Age (year old) ^a^	74 (69–80)	75 (70–78)	0.677
Pre-old	43 (53.8)	31 (50.0)	0.657
Old	37 (46.3)	31 (50.0)	
Body mass index (kg/m^2^) ^a^	23 (20–26)	23 (21–25)	0.761
History of ADEs	17 (21.3)	7 (11.3)	0.116
Medication information			
OTC drug use	22 (27.5)	20 (32.3)	0.538
Prescription records	75 (93.8)	56 (90.3)	0.534 ^b^
ODP dispensing	15 (18.8)	3 (4.8)	0.020 ^b^
No. of prescribed oral medications ^a^	7 (5–9)	4 (2–5)	<0.001
Non-polypharmacy	17 (21.3)	34 (54.8)	<0.001
Polypharmacy	63 (78.7)	28 (45.2)	
No. of consulting medical institutions			
One institution	45 (56.3)	45 (72.6)	0.045
Two or more institutions	35 (43.8)	17 (27.4)	

^a^ Median (IQR). ^b^ Fisher’s exact test. PIM, potentially inappropriate medication; ADE, adverse drug event; OTC, over the counter; ODP, one-dose package; IQR, interquartile range.

**Table 5:** Univariate and multivariable analyses results for a VAS score of 100% (*n* = 142).

	Univariate Analysis			Multivariable Analysis	
	VAS Score = 100%	VAS Score < 100%	*p*-Value	OR (95% CI)	*P*-Value
	(*n* = 66)	(*n* = 76)			
Patient characteristics					
Female sex	31 (47.0)	24 (31.6)	0.060	1.70 (0.84–3.47)	0.139
Age (year old) ^a^	75 (70–79)	74 (70–79)	0.946		
Pre-old	33 (50.0)	41 (54.0)	0.639	1	
Old	33 (50.0)	35 (46.1)		1.26 (0.60–2.66)	0.542
Body mass index (kg/m^2^) ^a^	23 (21–26)	23 (21–25)	0.609		
History of ADEs	11 (16.7)	13 (17.1)	0.945	0.802 (0.31–2.05)	0.644
Medication information					
OTC drug use	23 (34.9)	19 (25.0)	0.200		
Prescription records	63 (95.5)	68 (89.5)	0.221		
ODP dispensing	8 (12.1)	10 (13.2)	0.853		
No. of prescribed oral medications a	6 (3–8)	5 (3–7)	0.300		
Non-polypharmacy	22 (33.3)	29 (38.2)	0.550	1	
Polypharmacy	44 (66.7)	47 (61.8)		1.33 (0.59–3.04)	0.491
No. of consulting medical institutions					
One institution	39 (59.1)	51 (67.1)	0.323	1	
Two or more institutions	27 (40.9)	25 (32.9)		1.19 (0.55–2.57)	0.657
No. of patients with PIM use	45 (68.2)	35 (46.1)	0.008	2.58 (1.20–5.72)	0.015

^a^ Median (IQR). VAS, visual analogue scale; OR, odds ratio; CI, confidence interval; ADE, adverse drug event; OTC, over the counter; ODP, one-dose package; PIM, potentially inappropriate medication; IQR, interquartile range.

## Supplementary Materials

The following are available online at https://www.mdpi.com/1660-4601/17/16/5940/s1, Table S1: List of drug category, class, target patient population, and recommendation for PIMs screening in this study.
