# Supplementary material for: Association of Self-Reported Medication Adherence with Potentially Inappropriate Medications in Elderly Patients: A Cross-Sectional Pilot Study
Source: Int J Environ Res Public Health. 2020 Aug 16;17(16):5940. doi: 10.3390/ijerph17165940 (PMC7460224; doi:10.3390/ijerph17165940)
Supplement: Supplementary file 1 [file ijerph-17-05940-s001.pdf]

Table S1. List of drug category, class, target patient population, and recommendation for PIMs screening in this study.

Category	Class	Target patient population	Recommendation
Hypnotics	Benzodiazepines	All	Avoid
	Nonbenzodiazepines	All	Avoid
Sulpiride	Sulpiride	All	If used, 50 mg/day or less
Antithrombotic drugs [a]	Antithrombotic drugs (combination)	All	Do not use for a long period (12 months or more) and use it alone.
Diuretics [b]	Loop diuretic	All	If the decrease in circulating plasma volume is suspected, the use should be minimized, and discontinuation or dose reduction should be considered.
Alpha blocking agents	Alpha 1 blocking agents	All	Avoid
H2 receptor antagonists	H2 receptor antagonists	All	Avoid
Laxative	Magnesium oxide	Patients with renal dysfunction	Avoid
Antidiabetes drugs	Sulfonylurea	All	Avoid
	Biguanide	All	Avoid
	Thiazolidine	Patients with heart failure	Avoid
	SGLT2 inhibitor	All	Avoid
Overactive bladder drugs	Muscarinic receptor antagonist	All	Avoid
NSAIDs	NSAIDs	All	Consideration of combined use of PPIs to prevent gastrointestinal adverse events in patients with difficult discontinuation.

PIM, potentially inappropriate medication; SGLT, Sodium-glucose co-transporter; NSAIDs, nonsteroidal anti-inflammatory drugs.

[a] We could not obtain accurate data on the long-term combined use of multiple antithrombotic agents in this study. Thus, the recommended administration period of the combined use of multiple antithrombotic agents was excluded from screening.

[b] This item was judged by a pharmacist and a doctor other than the attending physician.
